# Enzootic transmission of *Leishmania* spp. in gallery forests of the Brazilian Cerrado

**DOI:** 10.1590/S1984-29612024073

**Published:** 2024-12-06

**Authors:** Aline Rapello, Andrey José de Andrade, Nadjar Nitz, Thaís Tâmara Castro Minuzzi-Sousa, Tamires Emanuele Vital, Tauana de Sousa Ferreira, Douglas de Almeida Rocha, Marcos Takashi Obara, Renata Velôzo Timbó, Jônatas Barbosa Cavalcante Ferreira, Rodrigo Gurgel-Gonçalves

**Affiliations:** 1 Programa de Pós-Graduação em Medicina Tropical, Núcleo de Medicina Tropical, Faculdade de Medicina, Universidade de Brasília – UnB, Brasília, DF, Brasil; 2 Departamento de Patologia Básica, Universidade Federal do Paraná – UFPR, Curitiba, PR, Brasil; 3 Laboratório Interdisciplinar de Biociências, Faculdade de Medicina, Universidade de Brasília – UnB, Brasília, DF, Brasil; 4 Pesquisador independente, Brasília, DF, Brasil; 5 Faculdade de Ceilândia, Universidade de Brasília – UnB, Brasília, DF, Brasil; 6 Laboratório de Parasitologia Médica e Biologia de Vetores, Faculdade de Medicina, Universidade de Brasília – UnB, Brasília, DF, Brasil

**Keywords:** Leishmaniasis, sylvatic cycles, rodents, opossums, phlebotominae, savanna, Leishmaniose, ciclos silvestres, roedores, gambás, flebotomíneos, savanas

## Abstract

Gallery forests harbor mammals and sand flies that may be involved in the transmission of *Leishmania* spp. parasites. Characterizing the enzootic cycles of *Leishmania* spp. is essential for understanding its transmission dynamics. We analyzed the presence of *Leishmania* spp. in mammals and sand flies in gallery forests during the dry season in the Cerrado. Four gallery forests were investigated in May and September 2014. Our capture effort included 1,280 HP trap-nights, 16 Shannon trap-nights for sand flies, and 5,120 trap-nights for mammals. After identifying the mammalian and sand fly species, SSU rRNA and ITS-1 polymerase chain reaction (PCR) were used to detect *Leishmania* spp. A total of 1,209 sand flies belonging to 13 species were captured, mainly *Bichromomyia flaviscutellata. Leishmania* spp. DNA was not detected in the analyzed sand fly females. PCR analysis of 153 mammals revealed *Leishmania* spp. in 20 samples (13%) in May (early dry season), when the infection rate was 31% in one gallery forest. The host species were *Rhipidomys macrurus*, *Gracilinanus agilis*, and *Didelphis albiventris*. We observed a low frequency of mammals infected with *Leishmania* spp., which was not detected in sand flies. Our results indicate that *Leishmania* spp. infection is higher in mammals during the early dry season in Cerrado gallery forests.

## Resumo

As matas de galeria abrigam mamíferos e flebotomíneos que podem estar envolvidos na transmissão de *Leishmania*. Caracterizar os ciclos enzoóticos de *Leishmania* spp. é essencial para entender a dinâmica da transmissão. Foi analisada a presença de *Leishmania* spp. em mamíferos e flebotomíneos em matas de galeria durante a estação seca no Cerrado. Quatro matas de galeria foram investigadas nos meses de maio e setembro. Nosso esforço de captura foi de 1.280 armadilhas-noite HP e 16 armadilhas-noite Shannon para flebotomíneos e 5.120 armadilhas-noite para mamíferos. Depois de identificar as espécies, realizamos PCRs direcionadas aos genes SSU rRNA e ITS-1 para detectar *Leishmania* spp. Capturou-se um total de 1.209 flebotomíneos pertencentes a 13 espécies, principalmente *Bichromomyia flaviscutellata*. Não foi detectado DNA de *Leishmania* spp. nas fêmeas de flebotomíneos analisadas. A PCR de 153 mamíferos revelou *Leishmania* spp. em 20 amostras (13%) em maio (início da estação seca), quando a taxa de infecção alcançou 31% em uma mata de galeria. Os hospedeiros pertenciam as espécies *Rhipidomys macrurus*, *Gracilinanus agilis* e *Didelphis albiventris*. Observou-se uma baixa frequência de mamíferos infectados por *Leishmania* spp., que não foi detectada em flebotomíneos. Os resultados indicam que a infecção por *Leishmania* spp. nos mamíferos é maior durante o início da estação seca nas matas de galeria do Cerrado.

## Introduction

Leishmaniasis is a parasitic zoonosis transmitted by phlebotomine sand flies and constitutes a neglected public health problem worldwide (Alvar et al., 2012; Burza et al., 2018). *Leishmania* spp. parasites infect a variety of hosts, including canids, opossums, rodents, bats, sloths, armadillos, and primates, all of which can serve as reservoirs in environments associated with human activity (Roque & Jansen, 2014). Enzootic transmission of *Leishmania* is known in different ecosystems in the Americas, from arid to extremely humid areas. Hence, characterization of the enzootic cycles is important to better understand the epidemiology of leishmaniasis (Cardoso et al., 2015; Silva et al., 2016; Shaw, 2019; Achilles et al., 2021).

Host exposure to *Leishmania* spp. in the wild may be associated with environmental and climate change, as well as labor and ecotourism activities (Oryan & Akbari, 2016). Approximately 140 species of potential hosts of *Leishmania* spp. are present in the Americas, of which approximately 60 may act as competent hosts (Glidden et al., 2023). *Leishmania* spp. have complex transmission cycles with specific epidemiological characteristics in each region where they occur (Ashford, 1996; Roque & Jansen, 2014; Lourenço et al., 2018; Achilles et al., 2021; Torres et al., 2024). The Cerrado gallery forests may contain potential reservoirs and vectors of *Leishmania* spp. parasites that contribute to the maintenance of enzootic transmission. Species of mammals and sand flies infected with *Leishmania* spp. have been identified in Cerrado biome (Almeida et al., 2015; Cardoso et al., 2015; Tonelli et al., 2017; Barrios et al., 2020; Brandão et al., 2020; Torres et al., 2024). However, the dynamics of *Leishmania* spp. transmission between sand flies and mammals in Cerrado gallery forests are poorly documented.

Several species of rodents and marsupials have been identified as potential reservoirs of *Leishmania* spp. in the Cerrado (Roque & Jansen, 2014). In the Federal District of Brazil (FD), Cardoso et al. (2015) identified six species of wild mammals that could be involved in the enzootic transmission of *Leishmania* spp. These species included *Gracilinanus agilis*, *Necromys lasiurus*, and *Rhipidomys macrurus*. The gallery forests of the Cerrado also contain sand fly species that can transmit *Leishmania* spp., such as *Nyssomyia whitmani* (Ferreira et al., 2014; Almeida et al., 2015; Rapello et al., 2018). Understanding the distribution, ecology, and infection rates of phlebotomine sand flies is essential for the preparation of preventive methods against leishmaniasis. However, in the Cerrado gallery forests, this knowledge is still emerging (Ferreira et al., 2014; Rapello et al., 2018). Data on *Leishmania* spp. in sand flies in the FD could indicate areas of transmission risk. Cases of cutaneous leishmaniasis have been reported in the FD since the 1980s (Sampaio & Paula, 1999). Since the first report of visceral leishmaniasis in the FD (Carranza-Tamayo et al., 2010), new human and canine cases have been recorded (Sampaio et al., 2009; Barbosa et al., 2022; Silva et al., 2023; Secretaria de Saúde do Distrito Federal, 2023).

The transmission cycles of *Leishmania* spp. in gallery forests are very diverse, considering the presence of at least three *Leishmania* species (*L. braziliensis*, *L. infantum*, *L. amazonensis*) that may be associated with several mammalian and phlebotomine species in specific microhabitats or periods. Therefore, to better understand the enzootic cycle dynamics of these parasites, it is important to conduct studies on both potential reservoirs and vectors in the same area at different times of the year. This information is often overlooked, because most studies focus on either sand fly vectors or mammalian reservoirs separately (Brito et al., 2009; Tonelli et al., 2017; Barrios et al., 2020; Brandão et al., 2020; Courtenay et al., 2023; Ratzlaff et al., 2023; Shaw et al., 2023; Silva et al., 2022; Torres et al., 2024). Therefore, our study aimed to investigate the enzootic transmission of *Leishmania* spp. in the gallery forests of the FD by simultaneously analyzing the natural infection of mammals and sand flies in different areas and periods of the year.

## Material and Methods

### Study areas

**Figure 1:**
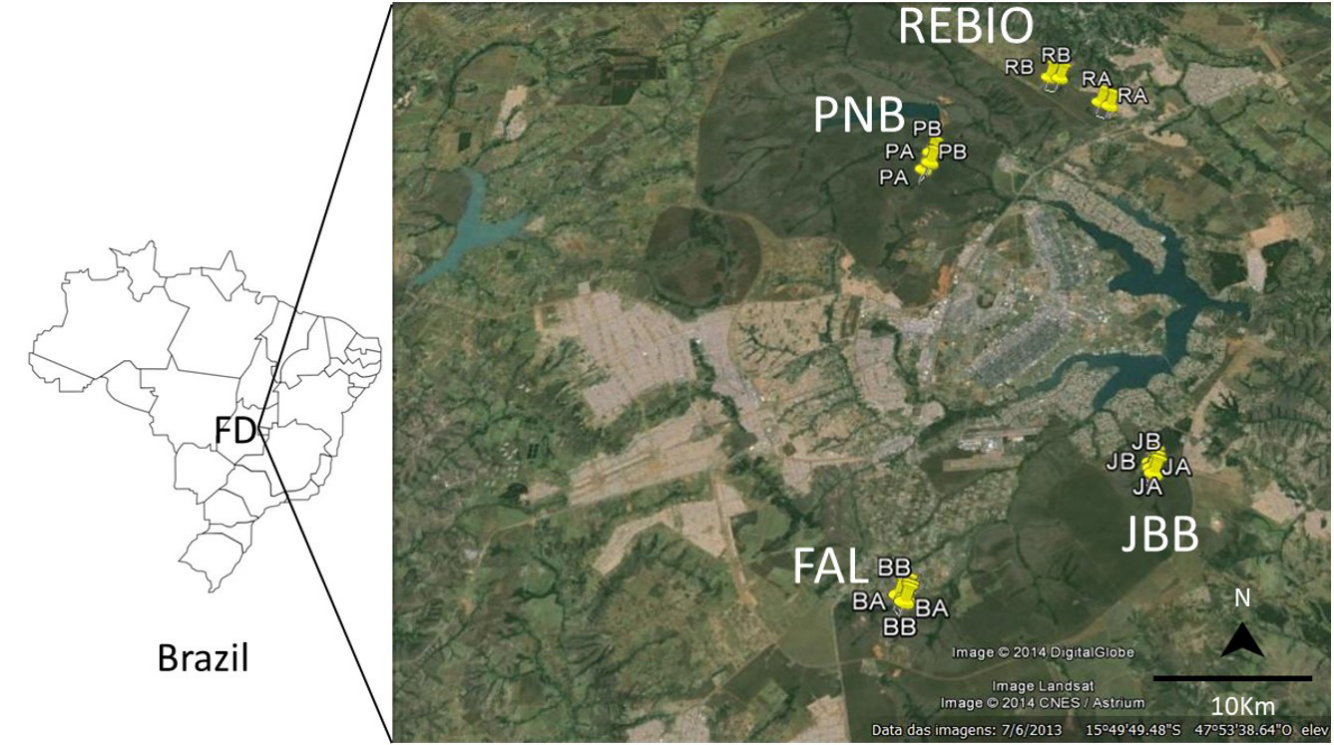
Location of the four study areas in the Federal District (FD). Yellow pins show the points within each transect. PNB: Brasilia National Park; REBIO: Contagem Biological Reserve; FAL: UnB's Água Limpa Farm; JBB: Brasilia Botanical Garden. Source: Google earth, 2014.

**Figure 2:**
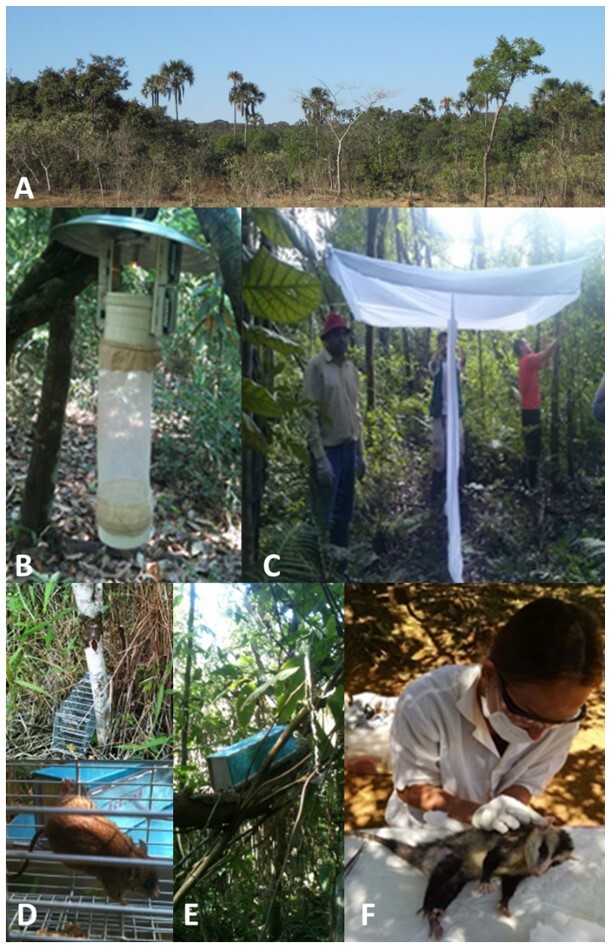
Sampling methods for sand flies and small mammals in gallery forests. A: Aspect of Cerrado gallery forest. B: HP trap for catching sand flies. C: Shannon-type trap. D: Tomahawk trap located on the ground and rodent captured. E. Sherman trap located in the tree layer. F. Mammal tissue sample collection procedure.

This study was conducted in four gallery forest areas close to urban settings (Figures 1 and 2A). Brasilia National Park – PNB (15°41′51″S, 047°57′32W) and Contagem Biological Reserve – REBIO (15°40′33 S, 047°51′45″ W) are in administrative regions in the North of the FD, where a higher number of canine visceral leishmaniasis cases (CVL) has been recorded since 2015 (Carranza-Tamayo et al., 2010; Secretaria de Saúde do Distrito Federal, 2023). The two others, UnB's Água Limpa Farm – FAL (15°57′17″ S, 047°58′30″W) and Brasilia Botanical Garden – JBB (15°53′17″ S, 047°50′33″ W), are located further south in the FD where a lower number of CVL cases was recorded (Cardoso et al., 2015; Barbosa et al., 2022; Silva et al., 2023; Secretaria de Saúde do Distrito Federal, 2023). The areas were sampled in May and September 2014, periods considered to be the early and late dry season in the Cerrado (Klink & Machado, 2005). The gallery forests were near areas where human and canine cases of leishmaniasis have been recorded. The climate of the region is tropical, with a cold and dry season (April–September) and a warm and rainy season (October–March). The average annual rainfall is approximately 1,600 mm, and the average annual temperature ranges between 18 and 20 °C. The detailed characteristics of the study areas have been previously described (Rapello et al., 2018).

### Sand fly sampling

In each gallery forest, two 1-km transects were determined, and 20 HP light traps per transect were installed in May and September 2014, as described by Rapello et al. (2018) with a list of sand flies in our study area. The HP traps were placed 1.5 m above the soil, spaced 50 m apart, and left for four consecutive days each month. Additionally, a Shannon trap was set 50 m away from each trapping transect (Figures 2 B and C). The heads and genitalia of the captured sand flies were dissected, clarified, and mounted on Canada balsam for species identification. The other body parts of the females (thorax, legs, and abdomen) were placed in microtubes with 1× phosphate-buffered saline (one specimen for each labeled tube) and stored at -20 ºC. For DNA extraction, the female sand flies (Supplementary Material file 1) were pooled by species into microtubes (Ferreira et al., 2015) and macerated with previously autoclaved plastic pistils.

### Small mammal sampling

Small mammals were captured on the same trails as those of the sand flies and during the same period using Tomahawk (17 × 17 × 52 cm) and/or Sherman (8 × 9 × 25 cm) live traps of size and strength proportional to the target species (Figures 2 D and E). The traps were alternately distributed along the same transect as those of the phlebotomine traps in the selected areas. The traps were spaced approximately 10 m apart at ground level and at a height of one to two meters when the habitat was vertically stratified, so that the tree layer could also be sampled in places conducive to the presence of small arboreal and scansorial mammals, such as liana nets and tree branches (Figure 2E). The traps were left open for four consecutive nights from approximately 8 to 8 a.m. the following day. The capture-mark-recapture method has been used to monitor small mammals (Cardoso et al., 2015). Mammals were marked with ear punchings. A mixture of sardine pieces, corn meal, canned corn, peanut paste, and mashed bananas was used as bait. After capture, the animals were taken to a base established by the team, where they were weighed, anesthetized for the collection of biological material (ear biopsy using scissors), and released back into the wild on the same day of capture (Figure 2F).

The anesthesia protocol used a combination of tiletamine/zolazepan-Zoletil 50^TM^ at a dose of 20–40 mg/kg, intramuscularly administered, or with 80 mg/kg ketamine hydrochloride and 10 mg/kg xylazine hydrochloride, depending on the species. Mammals were identified using taxonomic keys (Bonvicino et al., 2008). Skin fragments (ear tips) were collected, placed in dry Eppendorf tubes, and labeled.

The samples were transported to the Laboratory of Medical Parasitology and Vector Biology at the Faculty of Medicine, University of Brasilia. The samples collected in May were kept in a freezer at -20 °C for four months and, together with the samples collected in September, were transferred to a freezer at -80 °C for two months until the samples were processed.

### Molecular procedures

The DNA was extracted from the mammals and sand flies using the GE Healthcare Illustra™ Tissue & Cells Genomic Prep Mini Spin Kit according to the manufacturer’s recommendations. After extraction, the DNA was stored at -20 ºC. To determine the quality of the extracted DNA of sand flies, the cacophony gene of the IVS6 region of Phlebotominae was detected using specific primers (Llcac: 5'-GTGGC-CGAACATAATGTTAG-3' and Llcac 5'-CCACGAACAAGT-TCAACATC-3') with the aid of previously described reaction conditions (Machado et al., 2017).

Trypanosomatids were detected in the samples by nested PCR targeting the SSU rDNA gene. The first PCR was performed using S4 (5'-GAT CCA GCT GCA GGT TCA CC-3') and S12 (5'-GGT TGA TTC CGT CAA CGG AC-3') primers, generated a fragment of 520 base pairs (Uliana et al., 1994). The reactions were optimized with a final volume of 25 µL and the following standard mix: 100 ng of DNA, 2.5 μL of 10× amplification buffer (Invitrogen - Life Technologies, Brazil), 0.2 µM dNTPs (GE Healthcare), 0.75 µM of each primer, and 0.3 μL (1.5 U) Taq polymerase (Invitrogen-Life Technologies, Brazil). For the nested PCR, 2 µL of S4/S12 PCR products were reamplified using S17 (5'-CCA AGC TGC CCA GTA GAAT-3') and S18 (5'-TCG GGC GGA TAA AAC CC-3') internal primers, which amplify a region of 490 base pairs (Savani et al., 2009). The PCR was performed as described above. The amplification was performed using a MyCycler thermal cycler. The PCR cycle was 94 °C for 3 min, followed by 35 cycles of 94 °C for 1 min, 58 °C for 1 min, 72 °C for 1 min; 3 cycles of 94 °C for 1 min, 58 °C for 1 min, 72 °C for 1 min; 72 °C for 1 min, and a final extension at 7 2°C for 7 min. Negative (blank) and positive controls included DNA extracted from the reference strains of *Leishmania braziliensis* (MHOM/BR/75/M2903) and *Leishmania infantum* (MHOM/BR/74/PP75).

To determine the *Leishmania* infection rate of sand flies and mammals collected from the gallery forests, DNA samples were analyzed using the *Leishmania* ITS-1 primers (LITSR: 5'-CTGGATCATTTCCGATG-3' and LITSF: 5'-TGATACCACTTATCGCACTT-3'), which amplifies a fragment of approximately 265 base pairs (Schönian et al., 2003) The reaction was prepared for a final volume of 25 µL containing 100 ng of DNA template, 2.5 µL of 10× amplification buffer (Invitrogen - Life Technologies, Brazil), 0.2 µM dNTP mix (GE Healthcare), 0.25 µM of each primer (LITSR and LITSF), and 0.3 µl (1.5 U) of Taq DNA Polymerase (Invitrogen - Life Technologies, Brazil). Amplifications were performed in a MyCycler™ automated thermal cycler using the following cycle: initial denaturation at 95 ºC for 2 min, followed by 40 repetitions of: denaturation at 95 ºC for 30 s, annealing at 57 ºC for 30 s, and extension at 72 ºC for 30 s. The final extension step was performed at 72 ºC for 5 min. Negative (blank) and positive controls included DNA extracted from the reference strains of *L. braziliensis* (MHOM/BR/75/M2903) and *L. infantum* (MHOM/BR/74/PP75).

The PCR products were analyzed using horizontal electrophoresis on a 1.3% agarose gel stained with an ethidium bromide solution (0.5 µg/mL). The amplicons were visualized under ultraviolet light, and the gels were photographed using an Eagle Eye system (Stratagene, La Jolla, USA).

### Statistical analysis

Fisher’s exact test was used to compare the proportions of infected animals between the northern and southern areas studied, the proportions of individuals positive for *Leishmania* spp. between the species, age and sex of the mammals and between the months studied using the software GraphPad InStat version 3 (InStat, 1998). We also calculated frequencies and proportions of infected animals with confidence intervals (Wilson binomial with a 95%score) (Newcombe, 1998) using the “Hmisc” package in the R 4.2.1 computer software, together with the RStudio 2023.03.1.446 interface.

## Results

### *Leishmania* was not detected in the sand flies.

The total capture effort was 1,280 HP trap-nights (160 per area each month) and 16 Shannon traps-nights (2 per area each month). In total, 1,209 sand flies were captured as described in Rapello et al. (2018), of which 594 were females belonging to 13 species. Of the 594 captured phlebotomine females, 580 were tested by PCR. The remaining 14 specimens were not tested because of damage during collection and/or dissection, or because they were stored in a species bank. Most species and females were recorded in FAL (10 spp., n=263) and REBIO (5 spp., n=270). At PNB, 44 females (4 spp.) and at JBB, only 4 females (3 spp.) were examined. The females that underwent molecular analyses were grouped into 87 pools (Supplementary Material File 1). The cacophony gene fragments were successfully amplified in all samples, indicating the quality of the DNA. However, ITS-1 PCR did not detect the expected product in any of the specimens. PCR targeting a region of the SSUrDNA gene also failed to detect positive samples. No evidence of trypanosomatid DNA was found in any of the female sand flies examined.

### *Leishmania* was detected in marsupials and rodents at the early dry season.

**Figure 3:**
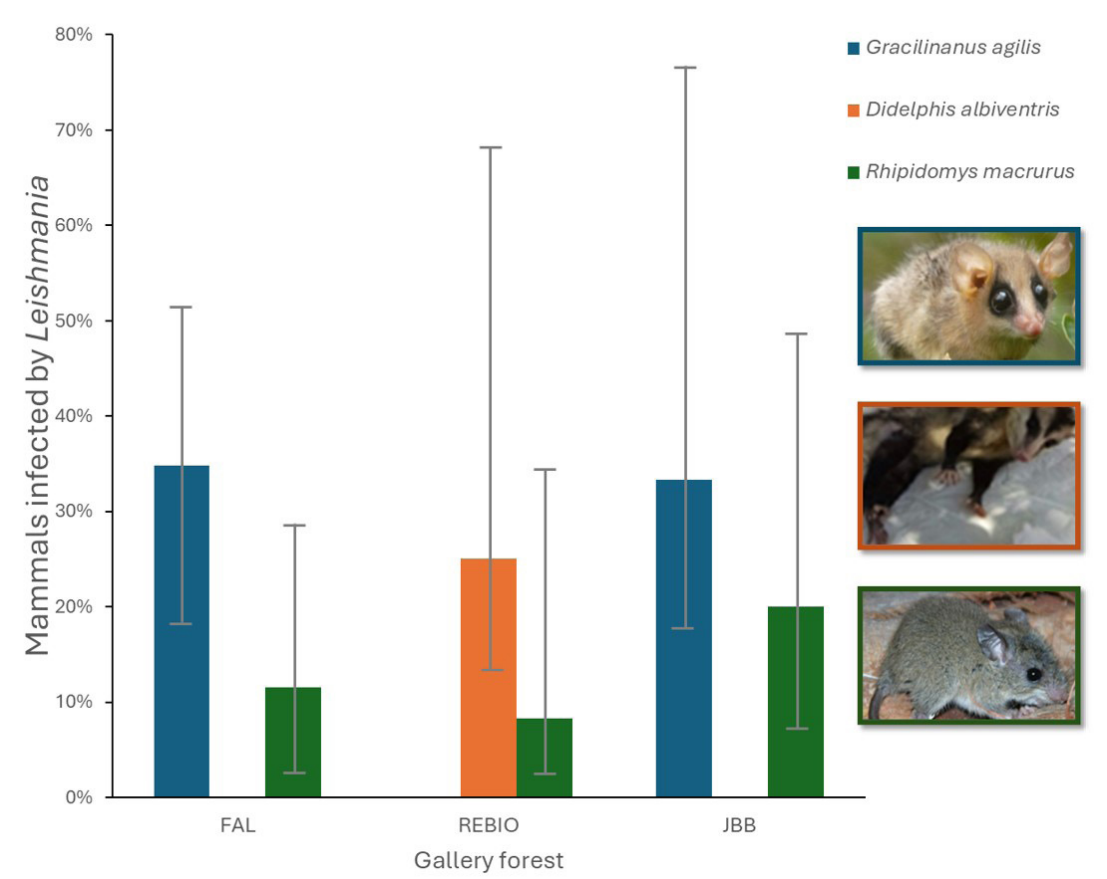
Proportions of mammals infected by *Leishmania* in the early dry season, 2014, in the study areas. The colors differentiate three species. The caption indicates the study areas: FAL: Água Limpa Farm, REBIO: Contagem Biological Reserve, JBB: Brasília Botanical Garden. The bars represent the 95% confidence intervals.

A total of 172 small mammals were captured during a 5,120-trapping night effort. Captures were mostly made during the early dry season (Table 1) and included six rodent species and three marsupial species. Ear fragments could not be collected from 19 animals because of natural mutilations. PCR analysis was performed on 153 ear fragments. The results showed that 20 (13.07%, 95% CI: 8.60–19.32) of the samples were positive for *Leishmania* spp. infection. Twelve samples were positive for both molecular markers, four samples amplified SSU DNA only, and the other four samples were positive for ITS-1 only (Supplementary Material File 2). The mammals infected with *Leishmania* spp. were identified as *Rhipidomys macrurus*, *Gracilinanus agilis,* and *Didelphis albiventris* (Figure 3). No statistically significant differences were found between the proportion of positive rodents and marsupials (Fisher, p>0.05) or between the proportion of positive mammals in the northern (REBIO and PNB) and southern (FAL and JBB) areas (Fisher, p>0.05). Similarly, no significant differences were observed between the proportions of positive males and females (Fisher, p>0.05), or between the proportions of positive juveniles and adults (Fisher, p>0.05). However, a statistically significant difference was observed in the proportion of positive mammals between the early and late dry seasons (Fisher, p <0.05). Considering only the gallery forests where positive mammals were detected, it was observed that the rodent *R. macrurus* was infected in all three areas and that the highest infection frequencies were recorded for *G. agilis* (Figure 3). The results indicated that *Leishmania* spp. infected marsupials and rodents in the studied gallery forests during the early dry season with frequencies ranging from 8% to 34%. No skin lesions were observed in infected mammals during the study.

**Table 1:** Distribution of mammal species captured in the study areas in May (early dry season) and September (late dry season) in gallery forests of the Federal District of Brazil, 2014.

**Species**	**FAL**			**REBIO**			**PNB**			**JBB**			**Tot**
	**M**	**S**	**Tot**	**M**	**S**	**Tot**	**M**	**S**	**Tot**	**M**	**S**	**Tot**	
*Gracilinanus agilis*	23	13	36	1	8	9	2	2	4	3	3	6	55
*Didelphis albiventris*	1	1	2	4	2	6	0	0	0	1	1	2	10
*Monodelphis americana*	0	1	1	0	1	1	0	0	0	0	0	0	2
*Rhipidomys macrurus*	26	5	31	12	6	18	0	2	2	10	4	14	65
*Necromys lasiurus*	0	0	0	0	0	0	2	0	2	1	0	1	3
*Nectomys rattus*	0	0	0	1	3	4	0	0	0	5	3	8	12
*Hylaeamys megacephalus*	0	1	1	4	0	4	0	0	0	0	0	0	5
*Oecomys bicolor*	0	3	3	1	2	3	1	0	1	3	2	5	12
*Calomys expulsus*	0	1	1	1	0	1	1	0	1	3	2	5	8
Total	50	25	75	24	22	46	6	4	10	26	15	41	172

FAL: Água Limpa Farm; REBIO: Contagem Biological Reserve; PNB: Brasília National Park; JBB: Brasília Botanical Garden; M: May; S: September; Tot: Total.

## Discussion

We observed a low frequency of mammals infected with *Leishmania*, which was not detected in sand flies. These findings suggest that enzootic transmission persists despite the absence or low frequency of *Leishmania* spp. infection in sand flies. The rodent *R. macrurus* was found to be infected in three gallery forests. Additionally, the most frequent infection was found in the marsupial *G. agilis*. Our results indicate that *Leishmania* infection is higher in mammals during the early dry season in the Cerrado gallery forests.

The rodent *R. macrurus* and marsupials *G. agilis* and *D. albiventris* were infected with *Leishmania* in gallery forests. Cardoso et al. (2015) detected *L. braziliensis* on the skin of one *N. lasiurus* and two *R. macrurus* using ITS1 PCR and sequencing. Our study areas were close to urban agglomerations where enzootic cycles of *L. braziliensis* occur (Cardoso et al., 2015); therefore, it is important to characterize their dynamics to better assess the risk of transmission to people living around these Cerrado gallery forests. The four samples identified as trypanosomatid DNA that were negative for *Leishmania* spp. DNA suggest the presence of other trypanosomatid species infecting *G. agilis* and *R. macrurus* in REBIO, JBB, and FAL in Brasília. Cardoso et al. (2015) discovered that *N. lasiurus* was infected with *Trypanosoma otospermophili* in both skin and blood samples, whereas *G. agilis* was found to be positive for *T. grosi* in a skin sample that was analyzed using the 24α rDNA marker and subsequent sequencing. These findings suggest that parasites, other than *Leishmania*, infect small mammals during enzootic cycles in Brasília.

No statistically significant difference was detected in the proportion of rodents and marsupials that tested positive in molecular tests. Moreover, the difference in the proportion of mammals infected with *Leishmania* spp. in the northern and southern regions was not statistically significant. To our knowledge, this is the first report of wild mammals testing positive for *Leishmania* spp. in an FAL gallery forest. The area commonly used for scientific studies appears to have undergone some degree of degradation owing to the human presence. Additionally, the area is near neighboring condominiums and is frequented by domestic dogs that roam forests. Furthermore, the presence of synanthropic animals; a high number of phlebotomines, including species of medical interest (Ferreira et al., 2014; Rapello et al., 2018); and bats infected with trypanosomatids, including *Leishmania* spp. (Lourenço et al., 2018), indicate the existence of an enzootic cycle of *Leishmania* spp. in this area. This could have contributed to the expansion of leishmaniasis in the southern region of the FD. Our study reported the presence of *Leishmania* spp.-infected mammals in the JBB forest, indicating a wide distribution of enzootic cycles in the region. The infected species were the same as those found in the FAL. The JBB includes areas for recreational activities, children’s education, and flora and fauna research. However, this also has a degree of anthropogenic influence. Additionally, the park’s proximity to neighborhoods, such as Lago Sul and Jardim Botânico, facilitates the entry of domestic animals into the forests. Synanthropic animals, such as *D. albiventris* and sand flies, which are potential vectors for *Leishmania* spp., have been captured. Although *Leishmania* spp. were not detected in the latter, the presence of infected rodents and other marsupial species provides evidence of parasite circulation. The absence of infection in sand flies does not necessarily indicate the absence of enzootic transmission in the area, as phlebotomine infection rates are typically low.

Although we found nine mammal species in the gallery forests, our study revealed that only *R. macrurus*, *G. agilis*, and *D. albiventris* were infected with *Leishmania* spp. We also found sand flies, which are potential vectors of *Leishmania* spp. Mammal exposure to *Leishmania* spp. depends on environmental conditions that support parasite transmission (e.g., temperature and humidity), vector biology, and competence. In Brazil, several studies have shown that the occurrence of sand flies is higher during the rainy season, when higher temperatures and humidity are observed, which may favor the feeding activity of the species (Ferreira et al., 2014). In addition, a higher abundance of mammals is also observed during the rainy season (Carmignotto et al., 2014; Mares & Ernest, 1995) when there is a greater availability of fruits, seeds, and insects. However, during the peak of the rainy season, no sand flies were detected in the gallery forests of FD (Ferreira et al., 2014), because the heavy rains in the forests must have disturbed the breeding sites. Thus, our hypothesis is that after the rainy season, when the temperature is still high and the rains are not so intense, there are better conditions for enzootic transmission, considering that the populations of mammals and sand flies would be relatively stable, favoring interactions and transmission of *Leishmania* spp. After exposure, infection maintenance depends on parasite-host interactions (Lainson, 1988), and explaining why *Leishmania* spp. is present in a specific host and not in another host is challenging (Marinho-Júnior et al., 2023). For instance, temperature may boost infection at 30 °C for one species and have a negative effect at lower temperatures. Moreover, sand fly behavior, such as biting activity and microhabitat, may influence mammalian exposure to *Leishmania* spp. These factors indicate that the enzootic transmission is complex (Roque & Jansen, 2014).

Mammalian tissue samples tested with the ITS1 molecular marker showed a higher percentage of positivity (10.4%) in this study than those tested with the same marker by Cardoso et al. (2015), who found 4.5% positivity in animals from the Cerrado and gallery forests in the PNB and REBIO. According to the same authors, who also carried out molecular tests with blood samples, seven of eight tissue samples were positive for the ITS1 marker, but no individual was positive simultaneously in the blood and tissue samples. Cardoso et al. (2015) also used the 24Sα rDNA target, whose amplification is suggestive of *Leishmania* spp., and obtained 20.1% positivity in the blood and tissue samples, because this target is more sensitive than ITS1, which in turn is more specific. The same is true for the SSU marker, which is more sensitive but less specific than ITS1 (Saraiva et al., 2017). However, when ITS1-nested PCR was performed, the sensitivity of the test considerably increased, reaching that of other targets (Oliveira et al., 2011). Four animals tested positive for the SSU marker without confirmation of the genus *Leishmania* spp. expected by the ITS1 marker. The choice of each test considers many variables, such as the specific purpose of the diagnosis, type of sample from which the DNA will be extracted, and facilities and technical knowledge available (Cruz et al., 2013, Freitas-Lidani et al., 2014).

The occurrence of sand flies in gallery forests in the FD had already been reported by Rapello et al. (2018). In the present study we showed no evidence of infection in the sand fly species evaluated (Supplementary Material File 1), including *Ny. whitmani* and *Bi. flaviscutellata* which have been found infected in Brazil and are competent species for transmitting *Leishmania* spp. (Queiroz et al. 1994; Luz et al., 2000; Neitzke-Abreu et al. 2014; Machado et al., 2017; Ferreira et al., 2018; Carvalho et al., 2018). *Ny. whitmani* and *Bi. flaviscutellata* have been found near houses in the FD (Sampaio et al., 2009) indicating the need to expand the investigation of *Leishmania* spp. in these species in future studies. Although no sand flies were infected in our study, *Leishmania* spp. DNA was found in several phlebotomine species in other reports. Paiva et al. (2006) identified *L. infantum* and *L. amazonensis* in *Lu. longipalpis* (infection rate of 3.9%), whereas Saraiva et al. (2011) found *L. infantum* in *Lu. longipalpis* (19%), *Ny. whitmani* (3.8%), *Ev. termitophila* (33.3%), and *Ny. intermedia* (14.4%). Rocha et al. (2010) reported an infection rate of 1.4% for *L. brazilensis* in *Ny. intermedia* and 11.1% in *Micropygomyia ferreirana*. *Leishmania* (*Viannia*) spp. were detected in *Ny. neivai* with an infection rate of 0.22% (Oliveira et al., 2011). Dias et al. (2013) reported infection rates of 0.6% for *Pintomyia fischeri* and 0.3% for *Migonemyia migonei*. Saraiva et al. (2017) detected *L. infantum* and *L. braziliensis* DNA in *Lu. longipalpis* with an infection rate of up to 2.6%. The results of sand fly infections across different endemic Brazilian regions and species show that the infection rates are low in most situations. In silvatic foci, infection of sand flies by *Leishmania* spp. is generally not detected or is very low (Neitzke et al., 2008; Brilhante et al., 2021; Costa et al., 2021; Morelli et al., 2024) despite the high incidence of the disease in urban regions (Maia-Elkhoury et al., 2008; Harhay et al., 2011; Barbosa et al., 2022). Sand flies may be infected with other trypanosomatid species. Ferreira et al. (2015) analyzed 210 female sand flies from 13 species collected in Brasília and found *Blastocrithidia* spp. in *Ny. whitmani*, and *Trypanosoma* sp. in *Ev. evandroi*.

This study has two limitations. First, the animals were captured on eight days (four in each month) of an almost six-month season. Therefore, future work could extend the number of fieldwork days to include more sampling days in the same month and even include other dry and rainy months to determine whether the lack of *Leishmania* spp. detection in sand flies is a result of the sampling process or whether it is a pattern observed in the Cerrado gallery forests. Monthly sampling throughout the year is considered relevant for the detection of *Leishmania* spp. in phlebotomines because of their relatively short lifespan compared to that of mammals. Mammals can maintain the infection for a longer period than that of phlebotomines, making it easier to detect *Leishmania* spp. in rodents and marsupials. In this study, we observed a higher prevalence of infection in mammals during the early dry season. This suggests that transmission may have occurred during the previous rainy season, when sand flies had not yet been sampled. Our results demonstrate that biannual sampling is not an optimal approach for this type of study. Future studies should be conducted with monthly sampling throughout the year to maximize the likelihood of detecting *Leishmania* spp. in these insects, in areas where the circulation of this parasite among mammals has already been proven. Monthly sampling could also be adequate to: 1) elucidate the infection dynamics throughout the year, 2) monitor infection among recaptured mammals to test for infectiousness, and 3) compare different tissues (skin and internal organs) from sampled mammals to investigate the parasite load throughout the year. Another limitation of this study is that it did not identify the species of *Leishmania* in positive mammalian samples by sequencing the PCR products. However, studies by our team have already shown that *L. braziliensis* and *Leishmania amazonensis* circulate in the PNB (Cardoso et al., 2015). Thus, it is likely that these species are widely distributed in the Cerrado gallery forests, as already identified in other studies (Roque & Jansen, 2014).

The results showed a low frequency of mammals infected with *Leishmania* spp. and either an absence or a low rate of natural infection by this parasite in sand flies in the gallery forests of the Cerrado during the dry season. Our results also indicated that *Leishmania* spp. infection was higher in mammals during the early dry season in gallery forests. To gain a better understanding of the enzootic cycles of *Leishmania* spp., new studies with longitudinal designs should be conducted in areas with potential *Leishmania* spp. risk, particularly in areas near urban areas, where sand flies are present, and with monthly capture of sand flies in the wet and dry seasons.

## Supplementary Material

Supplementary material accompanies this paper.

Supplementary file 1. Molecular identification of the pools of female phlebotomine sandflies subjected to molecular testing by area, species, month of collection and number of individuals in each pool.

Supplementary file 2. Identification of mammals positive for trypanosomatids/Leishmania spp. acording SSU and ITS-1 markers, area, and month.

This material is available as part of the online article from https://doi.org/10.1590/S1984-29612024073

## References

[B001] Achilles GR, Kautzmann RP, Chagas HDF, Pereira-Silva JW, Almeida JF, Fonseca FR (2021). Presence of trypanosomatids, with emphasis on *Leishmania*, in Rodentia and Didelphimorphia mammals of a rural settlement in the central Amazon region. Mem Inst Oswaldo Cruz.

[B002] Almeida PS, Andrade AJ, Sciamarelli A, Raizer J, Menegatti JA, Hermes SCNM (2015). Geographic distribution of phlebotomine sandfly species (Diptera: Psychodidae) in Central-West Brazil. Mem Inst Oswaldo Cruz.

[B003] Alvar J, Vélez ID, Bern C, Herrero M, Desjeux P, Cano J (2012). Leishmaniasis worldwide and global estimates of its incidence. PLoS One.

[B004] Ashford RW (1996). Leishmaniasis reservoirs and their significance in control. Clin Dermatol.

[B005] Barbosa DS, Belo VS, Bezerra JMT, Figueiredo FB, Werneck GL (2022). Factors associated with *Leishmania infantum* infection in dogs from urban areas endemic for visceral leishmaniasis in Brazil. Res Vet Sci.

[B006] Barrios SPG, Pereira LE, Casaril AE, Infran OM, Fernandes WS, Oshiro ET (2020). Phlebotominae (Diptera: Psychodidae) and Biomes in the State of Mato Grosso do Sul, Brazil. J Med Entomol.

[B007] Bonvicino CR, Oliveira JA, D’Andrea PS (2008). Guia dos Roedores do Brasil: com chaves para gêneros baseadas em caracteres externos..

[B008] Brandão EMV, Xavier SCC, Rocha FL, Lima CFM, Candeias ÍZ, Lemos FG (2020). Wild and domestic canids and their interactions in the transmission cycles of *Trypanosoma cruzi* and *Leishmania* spp. in an area of the Brazilian Cerrado. Pathogens.

[B009] Brilhante AF, Lima L, Ávila MM, Medeiros-Sousa AR, Souza JF, Santos NP (2021). Remarkable diversity, new records and *Leishmania* detection in the sand fly fauna of an area of high endemicity for cutaneous leishmaniasis in Acre state, Brazilian Amazonian Forest. Acta Trop.

[B010] Brito MEF, Andrade MS, Mendonça MG, Silva CJ, Almeida EL, Lima BS (2009). Species diversity of *Leishmania (Viannia)* parasites circulating in an endemic area for cutaneous leishmaniasis located in the Atlantic rainforest region of northeastern Brazil. Trop Med Int Health.

[B011] Burza S, Croft SL, Boelaert M (2018). Leishmaniasis. Lancet.

[B012] Cardoso RM, Araújo NNSL, Romero GAS, Souza TTCM, Dietrich AG, Mendes JD (2015). Expanding the knowledge about *Leishmania* species in wild mammals and dogs in the Brazilian savannah. Parasit Vectors.

[B013] Carmignotto AP, Bezerra AMR, Rodrigues FHG (2014). Nonvolant small mammals from a southwestern area of Brazilian Cerrado: diversity, habitat use, seasonality, and biogeography. Therya.

[B014] Carranza-Tamayo CO, Carvalho MSL, Bredt A, Bofil MIR, Rodrigues RMB, da Silva AD (2010). Autochthonous visceral leishmaniasis in Brasília, Federal District, Brazil. Rev Soc Bras Med Trop.

[B015] Carvalho BM, Dos Santos TV, da R Barata I, Lima JAN, Silveira FT, Vale MM (2018). Entomological surveys of *Lutzomyia flaviscutellata* and other vectors of cutaneous leishmaniasis in municipalities with records of *Leishmania amazonensis* within the Bragança region of Pará State, Brazil. J Vector Ecol.

[B016] Costa GS, Pereira AM, Castro TS, de Paulo PFM, Ferreira GEM, Medeiros JF (2021). Sand fly fauna and molecular detection of *Leishmania* species and blood meal sources in different rural environments in western Amazon. Acta Trop.

[B017] Courtenay O, Marinho-Júnior JF, Brito MEF, Monteiro JFCLS, Shaw JJ, Brandão-Filho SP (2023). Incidence of human and free-ranging wild rodent infections with *Leishmania* (*Viannia*) *braziliensis*, aetiological agent of cutaneous leishmaniasis. Pathogens.

[B018] Cruz I, Millet A, Carrillo E, Chenik M, Salotra P, Verma S (2013). An approach for interlaboratory comparison of conventional and real-time PCR assays for diagnosis of human leishmaniasis. Exp Parasitol.

[B019] Dias ES, Michalsky ÉM, Nascimento JC, Ferreira EC, Lopes JV, Fortes-Dias CL (2013). Detection of *Leishmania infantum*, the etiological agent of visceral leishmaniasis, in *Lutzomyia neivai*, a putative vector of cutaneous leishmaniasis. J Vector Ecol.

[B020] Ferreira JBC, Macedo MA, Rocha DA, Ferreira TS, Obara MT, Gurgel-Gonçalves R (2014). Ocorrência de flebotomíneos (Diptera: Psychodidae) em matas de galeria no Distrito Federal, Brasil. EntomoBrasilis.

[B021] Ferreira TS, Minuzzi-Souza TTC, Andrade AJ, Coelho TO, Rocha DA, Obara MT (2015). Molecular detection of *Trypanosoma* sp. and *Blastocrithidia* sp. (Trypanosomatidae) in phlebotomine sand flies (Psychodidae) in the Federal District of Brazil. Rev Soc Bras Med Trop.

[B022] Ferreira TS, Timbó RV, Minuzzi-Souza TTC, de Almeida Rocha D, Neiva M, de Albuquerque Ribeiro J (2018). High molecular prevalence of *Leishmania* in phlebotomine sand flies fed on chicken blood in Brazil. Vet Parasitol.

[B023] Freitas-Lidani KC, Messias-Reason IJ, Ishikawa EAY (2014). A comparison of molecular markers to detect *Lutzomyia longipalpis* naturally infected with *Leishmania* (*Leishmania*) *infantum.*. Mem Inst Oswaldo Cruz.

[B024] Glidden CK, Murran AR, Silva RA, Castellanos AA, Han BA, Mordecai EA (2023). Phylogenetic and biogeographical traits predict unrecognized hosts of zoonotic leishmaniasis. PLoS Negl Trop Dis.

[B025] Harhay MO, Olliaro PL, Costa DL, Costa CHN (2011). Urban parasitology: visceral leishmaniasis in Brazil. Trends Parasitol.

[B026] InStat (1998). GraphPad Software..

[B027] Klink CA, Machado RB (2005). Conservation of the Brazilian Cerrado. Conserv Biol.

[B028] Lainson R (1988). Ecological interactions in the transmission of the leishmaniases. Philos Trans R Soc Lond B Biol Sci.

[B029] Lourenço JLM, Minuzzi-Souza TTC, Silva LR, Oliveira AC, Mendonça VJ, Nitz N (2018). High frequency of trypanosomatids in gallery forest bats of a Neotropical savanna. Acta Trop.

[B030] Luz E, Membrive N, Castro EA, Dereure J, Pratlong F, Dedet JA (2000). *Lutzomyia whitmani* (Diptera: Psychodidae) as vector of *Leishmania* (*V.*) *braziliensis* in Paraná state, southern Brazil. Ann Trop Med Parasitol.

[B031] Machado TDO, Minuzzi-Souza TTC, Ferreira TS, Freire LP, Timbó RV, Vital TE (2017). The role of gallery forests in maintaining phlebotominae populations: Potential *Leishmania* spp. vectors in the Brazilian savanna. Mem Inst Oswaldo Cruz.

[B032] Maia-Elkhoury ANS, Alves WA, Sousa-Gomes ML, Sena JM, Luna EA (2008). Visceral leishmaniasis in Brazil: trends and challenges. Cad Saude Publica.

[B033] Mares MA, Ernest KA (1995). Population and community ecology of small mammals in a Gallery Forest of Central Brazil. J Mammal.

[B034] Marinho-Júnior JF, Monteiro JFCLS, Sales de Carvalho AW, Carvalho FG, Paiva Cavalcanti M, Shaw J (2023). High levels of infectiousness of asymptomatic *Leishmania (Viannia) braziliensis* infections in wild rodents highlights their importance in the epidemiology of American Tegumentary Leishmaniasis in Brazil. PLoS Negl Trop Dis.

[B035] Morelli LC, Pita-Pereira D, Britto C, Araújo-Pereira T, Souza LAF, Germano KO (2024). *Leishmania (Leishmania) infantum* DNA detection in *Nyssomyia neivai* in Vale do Ribeira, Paraná, Brazil. Mem Inst Oswaldo Cruz.

[B036] Neitzke HC, Scodro RBL, Castro KRR, Sversutti ACD, Silveira TGV, Teodoro U (2008). Pesquisa de infecção natural de flebotomíneos por *Leishmania*, no Estado do Paraná. Rev Soc Bras Med Trop.

[B037] Neitzke-Abreu HC, Reinhold-Castro KR, Venazzi MS, Scodro RB, Dias AC, Silveira TG (2014). Detection of *Leishmania (Viannia)* in *Nyssomyia neivai* and *Nyssomyia whitmani* by multiplex polymerase chain reaction, in Southern Brazil. Rev Inst Med Trop São Paulo.

[B038] Newcombe RG (1998). Two-sided confidence intervals for the single proportion: comparison of seven methods. Stat Med.

[B039] Oliveira DM, Reinhold-Castro KR, Bernal MVZ, Oliveira Legriffon CM, Lonardoni MVC, Teodoro U (2011). Natural infection of *Nyssomyia neivai* by *Leishmania (Viannia*) spp. in the State of Paraná, Southern Brazil, detected by multiplex polymerase chain reaction. Vector Borne Zoonotic Dis.

[B040] Oryan A, Akbari M (2016). Worldwide risk factors in leishmaniasis. Asian Pac J Trop Med.

[B041] Paiva BR, Secundino NFC, Nascimento JC, Pimenta PFP, Galati EAB, Andrade HF (2006). Detection and identification of *Leishmania* species in field-captured phlebotomine sandflies based on mini-exon gene PCR. Acta Trop.

[B042] Queiroz RG, Vasconcelos IA, Vasconcelos AW, Pessoa FA, de Sousa RN, David JR (1994). Cutaneous leishmaniasis in Ceara state in northeastern Brazil: incrimination of *Lutzomyia whitmani* (Diptera: Psychodidae) as a vector of *Leishmania braziliensis* in baturite municipality. Am J Trop Med Hyg.

[B043] Rapello A, Andrade AJ, Rocha DA, Ferreira JCB, Timbó RV, Obara MT (2018). An updated list of sand flies (Diptera, Psychodidae, Phlebotominae) in the Federal District of Brazil. Check List.

[B044] Ratzlaff FR, Osmari V, Silva D, Vasconcellos JSP, Pötter L, Fernandes FD (2023). Identification of infection by *Leishmania* spp. in wild and domestic animals in Brazil: a systematic review with meta-analysis (2001–2021). Parasitol Res.

[B045] Rocha LS, Falqueto A, Santos CB, Ferreira AL, Graça GC, Grimaldi G (2010). Survey of natural infection by *Leishmania* in sand fly species collected in southeastern Brazil. Trans R Soc Trop Med Hyg.

[B046] Roque ALR, Jansen AM (2014). Wild and synanthropic reservoirs of *Leishmania* species in the Americas. Int J Parasitol Parasites Wildl.

[B047] Sampaio RNR, Gonçalves MC, Leite VA, França BV, Santos G, Carvalho MSL (2009). Study on the transmission of American cutaneous leishmaniasis in the Federal District. Rev Soc Bras Med Trop.

[B048] Sampaio RNR, Paula CDR (1999). Leishmaniose tegumentar americana no Distrito Federal. Rev Soc Bras Med Trop.

[B049] Saraiva L, Andrade JD, Falcão AL, Carvalho DAA, Souza CM, Freitas CR (2011). Phlebotominae fauna (Diptera: Psychodidae) in an urban district of Belo Horizonte, Brazil, endemic for visceral leishmaniasis: Characterization of favored locations as determined by spatial analysis. Acta Trop.

[B050] Saraiva L, Leite CG, Lima ACVMR, Carvalho LOA, Pereira AAS, Rugani JMN (2017). Seasonality of sand flies (Diptera: Psychodidae) and *Leishmania* DNA detection in vector species in an area with endemic visceral leishmaniasis. Mem Inst Oswaldo Cruz.

[B051] Savani ESMM, Nunes VLB, Galati EAB, Castilho TM, Zampieri RA, Floeter-Winter LM (2009). The finding of *Lutzomyia almerioi* and *Lutzomyia longipalpis* naturally infected by *Leishmania* spp. in a cutaneous and canine visceral leishmaniases focus in Serra da Bodoquena, Brazil. Vet Parasitol.

[B052] Schönian G, Nasereddin A, Dinse N, Schweynoch C, Schallig HDFH, Presber W (2003). PCR diagnosis and characterization of *Leishmania* in local and imported clinical samples. Diagn Microbiol Infect Dis.

[B053] Secretaria de Saúde do Distrito Federal (2023). Informes Epidemiológicos - Leishmaniose..

[B054] Shaw J (2019). The importance of understanding enzootic cycles in the epidemiology of zoonotic diseases with special reference to the American leishmaniases. Trans R Soc Trop Med Hyg.

[B055] Shaw JJ, Marinho-Júnior JF, Courtenay O, Brandão-Filho SP (2023). Assessing reservoir host status in leishmaniasis with special reference to the infectiousness of *Leishmania (Viannia) braziliensis* infections in wild rodents. Rev Soc Bras Med Trop.

[B056] Silva CJ, Monteiro JFCLS, Lima KPB, Silva CSAG, Almeida ÉL, Souza SF (2022). Study on the zoonotic cycle of tegumentary leishmaniasis in an endemic area of a metropolitan region in the Northeastern region of Brazil. Rev Inst Med Trop São Paulo.

[B057] Silva DM, Teixeira AIP, Romero GAS (2023). Socioeconomic status of guardians as a risk factor for canine visceral leishmaniasis: a cohort study in an endemic area of the Federal District, Brazil. Am J Trop Med Hyg.

[B058] Silva EM, Alves LC, Guerra NR, Farias MPO, Oliveira ELR, Souza RC (2016). *Leishmania* spp. in *Didelphis* spp. from Northeastern Brazil. J Zoo Wildl Med.

[B059] Tonelli GB, Tanure A, Rego FD, Carvalho GML, Stumpp R, Ássimos GR (2017). *Leishmania (Viannia) braziliensis* infection in wild small mammals in ecotourism area of Brazil. PLoS One.

[B060] Torres JM, Oliveira CE, Santos FM, Sano NY, Martinez ÉV, Alves FM (2024). Trypanosomatid diversity in a bat community of an urban area in Campo Grande, Mato Grosso do Sul, Brazil. Infect Genet Evol.

[B061] Uliana SRB, Nelson K, Beverley SM, Camargo EP, Floeter-Winter LM (1994). Discrimination amongst *Leishmania* by polymerase chain reaction and hybridization with small subunit ribosomal DNA derived oligonucleotides. J Eukaryot Microbiol.

